# ANNOUNCEMENTS & RESOURCES

**Published:** 2018-06-03

## 2018 ROP Africa Symposium

The International Pediatric Ophthalmology and Strabismus Council and The Department of Pediatric Ophthalmology at The Red Cross War Memorial Children's Hospital, Cape Town are proud to announce the 2018 ROP Africa Symposium.

**Figure:**
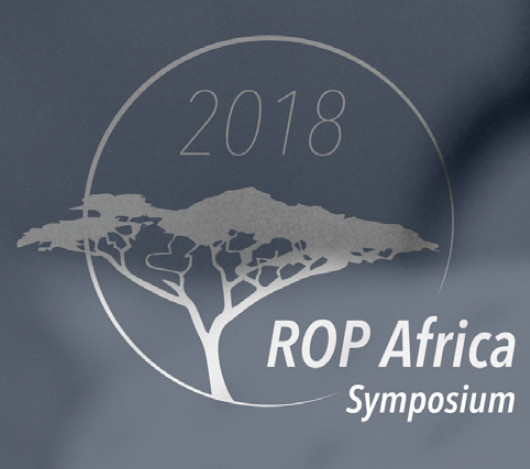


**Date:** 3–4 September 2018

**Venue:** Surgical Skills Training Centre, Red Cross War Memorial Children's Hospital, Cape Town, South Africa

Mark your calendars for this international 2-day clinical conference and hands-on workshop for ophthalmologists and neonatologists caring for infants with retinopathy of prematurity. For more details, contact: Jenny Baker at **jbaker9@uic.edu**

## Courses

### MSc Public Health for Eye Care, London School of Hygiene & Tropical Medicine

Fully funded scholarships are available for Commonwealth country nationals. The course aims to provide eye health professionals with the public health knowledge and skills required to reduce blindness and visual disability.

For more information visit **www.lshtm.ac.uk/study/masters/mscphec.html** or email **romulo.fabunan@lsthm.ac.uk**

#### Free online courses

**ICEH Open Education for eye care programme** offers a series of online courses in key topics in public health eye care. All the courses are free to access.

Courses:

Global Blindness, Eliminating Trachoma, Ophthalmic Epidemiology Basic Principles (1) and Application to Eye Disease (2).

More free courses coming! Certification also available.

For more information visit **http://iceh.lshtm.ac.uk/oer/**

## Subscriptions

Contact Anita Shah **admin@cehjournal.org**

### Subscribe to our mailing list

**web@cehjournal.org** or visit **www.cehjournal.org/subscribe**

### Visit us online

**www.cehjournal.org www.facebook.com/CEHJournal https://twitter.com/CEHJournal**

**World Retinoblastoma Awareness week**

To increase awareness about Retinoblastoma every year seven days from the second Sunday in May is celebrated as ‘World Retinoblastoma Awareness Week’, this year it will be celebrated from **May 13th to May 19th 2018**

